# Case of Partial Paralysis, Cured by Urtication

**Published:** 1821-09

**Authors:** John Piper


*Case of Partial Paralysis, cured by Urtication.* By Mr. JOHN PIPER, Assistant to Mr. THOMAS GEAST, Surgeon.

MISS B. of Great Hampton-street, in this town, about eighteen years of age, of a nervous temperament, very tall, slender, and delicate habit of body, described by her friends as having "outgrown her strength," had been upwards of five months afflicted with a total paralysis of the right arm, not having therein the least sense of feeling or capability of motion. The accession of the disease she believes to have been spontaneous, as she never recollects receiving any injury. It was marked by a considerable degree of pain and œdematous swelling of the hand and fingers, the extremities of which were of a livid hue, and the tendons became contracted; to obviate which, she was directed by her medical attendant to make use of a wooden frame, corresponding in size and shape with the hand, and retained thereto by a bandage. After some time the pain and tension subsided, and were succeeded by a gradual wasting and flaccidity of the muscular fabric, a death-like coldness, and almost complete insensibility; which, extending itself, affected the whole arm and its scapular appendages. Under these circumstances, she applied, successively, to six or seven eminent medical gentlemen, who had recourse to a multiplicity of means calculated to restore the suspended energy: amongst which were fomentations, frictions, and a variety of stimulating applications, to the limb and along the course of the vertebral column; topical bleedings, by leeches and cupping; vesicatories, electricity, galvanism, &c. &c.; which were severally persisted in for a long time, but without being productive of any beneficial results. She at length became weary of her treatment, and had declined making use of any farther attempts for the resuscitation of her arm, supposing that the whole powers of the healing art had been already exhausted.

On the 15th of July, 1820, being called upon to attend another member of the family, the case was related to me, merely by accident, not under any idea, I believe, of its being benefitted by my humble efforts: neither could I entertain the presumption of supposing that any agency of mine might be crowned with success, after the measures heretofore employed had been fruitlessly expended. Fortuitously the idea of urtication occurred to me, the proposition of which, I perceived, excited a risible propensity: however, on the same evening, a rod of nettles was procured, and its application immediately commenced with. The ensuing morning was ushered in with a repetition of the piquant flagellation, and continued from time to time through the day; each successive application evincing an increased development of sensibility.

On the morning of the 17th, my astonishment was very great when the patient came to me, with an air of exultation and gratitude, displaying her arm (which had hitherto been suspended as useless in a sling,) in every position, with all the freedom and agility of its pristine state. The long dormant faculties of sensation and motion were perfectly developed; no other trace of the affection remained but a slight weakness of the wrist, which was soon removed by a strengthening plaster and bandage.

It will be proper to observe, that this young lady had been affected for a long time with indigestion, hepatic derangement, and general nervous debility: her days were a series of inactive somnolency and unpleasurable reminiscences; at night, instead of receiving the salutary and refreshing influence of "tir'd nature's sweet restorer, balmy sleep," her pillow was haunted by incubi, and all the terrific vampire forms of a perturbed imagination. Her appetite was depraved; the sight of animal food was particularly loathsome, and her sole subsistence for many months was tea.

I commenced my medical measures with the exhibition of an emetic, composed of cupri sulphas and ipecacuanha, which was afterwards occasionally repeated.

℞. Pulveris rhei, ʒss.; Piperis cayanensis, ℈j.; Extracti gentianæ, q. s. ut fiunt pilul. No. xxiv. Capiat duas omni mane.

℞. Infusi cascarillæ, Misturæ camphoræ, āā, ℥iijss.; Ammoniæ carbonatis, ʒj.; Tinct. calumbæ; Sp. ætheris sulphurici, āā, ℥ss. ℳ. sumat cochlearia duo largater quotidie.

℞. Pilulæ hydrargyri, gr. v. Pilula alternis noctibus sumenda.

I directed the frequent use of the pediluvium, flannel to be worn next the body, and woollen stockings for the inferior extremities. The further indulgence in the use of tea was strictly prohibited, and instead of it was enjoined the most regular and nutritious diet, with a liberal allowance of sherry wine. A frequent change of air and scene, and agreeable exercise, was also recommended. My injunctions were willingly complied with, and rigorously persisted in; and, in a short space of time, I had the gratification to find effected in my patient that complete healthy condition of the system she had so long been a stranger to.

*Lionel-street, Birmingham; 19th July*, 1821.

